# A calcitonin-producing pancreatic neuroendocrine neoplasm treated with distal pancreatectomy a lengthy time after a left trisectionectomy for liver metastases: a case report

**DOI:** 10.1186/s40792-022-01575-7

**Published:** 2022-12-08

**Authors:** Ryusei Yamamoto, Ryuzo Yamaguchi, Katsushi Yoshida, Masataka Ando, Yoshitaka Toyoda, Aya Tanaka, Kenji Kato

**Affiliations:** Division of Surgery, Inazawa Municipal Hospital, 100 Nazukacho-Numa, Inazawa, Aichi 492-8510 Japan

**Keywords:** Calcification, Calcitonin, Hepatectomy, Neuroendocrine neoplasms, Pancreatectomy

## Abstract

**Background:**

Calcitonin-producing pancreatic neuroendocrine neoplasms (PanNENs) are extremely rare. There have been no reports of a patient in whom liver metastases were the presenting finding, and a calcitonin-producing PanNEN was subsequently detected after a lengthy period.

**Case presentation:**

A 53-year-old man had diarrhea for several years. Computed tomography (CT) revealed multiple liver tumors. We performed a left trisectionectomy with a bile duct resection. The histologic examination showed neuroendocrine tumors G1. Immunohistochemistry was positive for calcitonin and the serum calcitonin level was elevated. Neuroendocrine neoplasms of hepatic origin are extremely rare, so a systemic exploration was performed, but no tumor was detected. CT showed a 4-mm calcification in the pancreatic body, but no contrast-enhanced mass was noted. Although the liver tumors were resected, the diarrhea and high serum calcitonin level persisted. Serial examinations were performed for 6 years, but no tumor was identified; however, 6.5 years after the hepatectomy the serum calcitonin level increased. CT showed a 10-mm contrast-enhanced mass in the calcified area of the pancreatic body. A distal pancreatectomy was performed. The histologic examination showed a neuroendocrine tumor G1, which mimicked the liver tumors. Immunohistochemistry was positive for calcitonin. After the distal pancreatectomy, the serum calcitonin level decreased and diarrhea resolved. The calcitonin-producing neuroendocrine neoplasm was considered the pancreatic primary and the hepatic tumors were metastases.

**Conclusions:**

Calcitonin-producing PanNENs may be initially recognized as liver tumors and may become evident after a lengthy period, thus long-term observation is recommended. Aggressive surgeries may contribute to long-term survival.

**Supplementary Information:**

The online version contains supplementary material available at 10.1186/s40792-022-01575-7.

## Background

Pancreatic neuroendocrine neoplasms (PanNENs) are divided into functional and non-functional [1]. The majority of functional PanNENs are insulinomas followed by gastrinomas, and there are few glucagonomas, somatostatinomas, vasoactive intestinal polypeptide producing tumors, and the others [1]. However, functional calcitonin-producing PanNENs are extremely rare [2, 3].

Among patients with calcitonin-producing PanNENs, approximately 60% have distant metastases at the time of diagnosis, of which liver metastases are the most common [2]. There have been no reports of a patient in whom liver metastases were the presenting finding, and a calcitonin-producing PanNEN was subsequently detected after a lengthy period.

Surgical resection is the cornerstone for calcitonin-producing PanNENs with liver metastases, as in other PanNENs [2, 4]. However, the indication or optimal extent of surgical resection for calcitonin-producing PanNENs with liver metastases remains unclear.

Herein we report a patient with a calcitonin-producing PanNEN who was treated with a distal pancreatectomy a long time after undergoing a left trisectionectomy for liver metastases.

## Case presentation

**Fig. 1:**
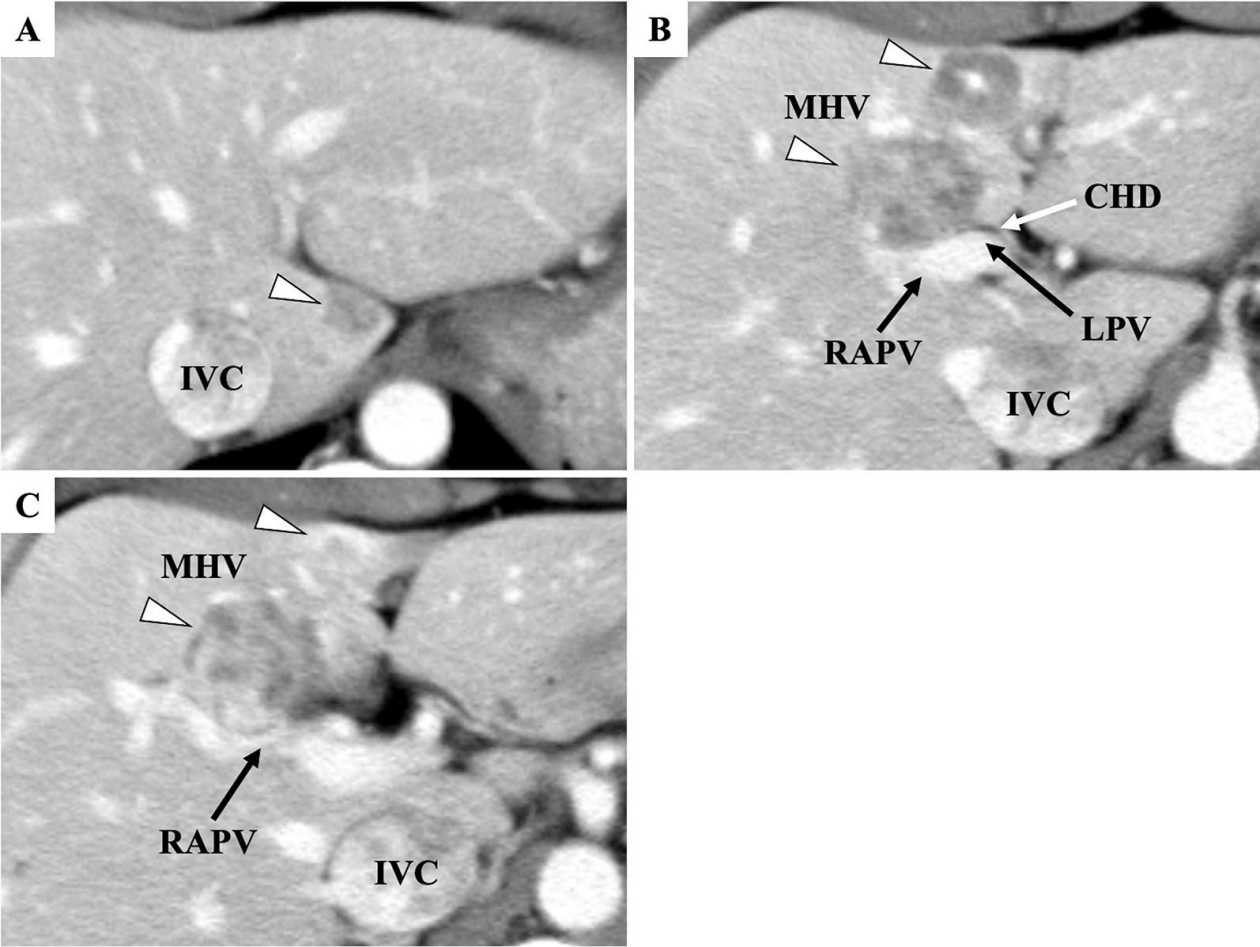
Computed tomography shows hypervascular tumors of the liver in segment 1 (**A**) and segment 4 (**B**). IVC, inferior vena cava; MHV, middle hepatic vein; LPV, left portal vein; RAPV, right anterior portal vein; CHD, common hepatic duct

A 53-year-old man was shown to have an elevated serum carcinoembryonic antigen level during a routine physical examination. His medical history was unremarkable, but he had diarrhea for several years. Computed tomography (CT) identified 3 hypervascular tumors in the liver, as follows: segment 1, 12 mm; and segment 4, 18 mm and 28 mm (Fig. 1). A percutaneous transhepatic biopsy of the tumor was performed, and an adenocarcinoma was suspected. We did not perform immunohistochemistry for distinguishing a neuroendocrine neoplasm. We diagnosed liver tumors as intrahepatic cholangiocarcinomas. A tumor of the segment 4 abutted to the right anterior portal vein, left portal vein, and common hepatic duct on CT, so we considered the right anterior and left portal veins resection and a bile duct resection to be necessary. The right posterior portal vein branched independently from the main portal vein, and was distant from the tumors. Moreover, a caudate lobectomy for a tumor of the segment 1 was also needed. Thus, we planned a left trisectionectomy with a bile duct resection. We performed a staging laparoscopy and portal embolization in preparation for a left trisectionectomy. Two weeks later, the remnant liver volume was 47.7% and the future liver remnant plasma clearance rate of indocyanine green was 0.106.

**Fig. 2:**
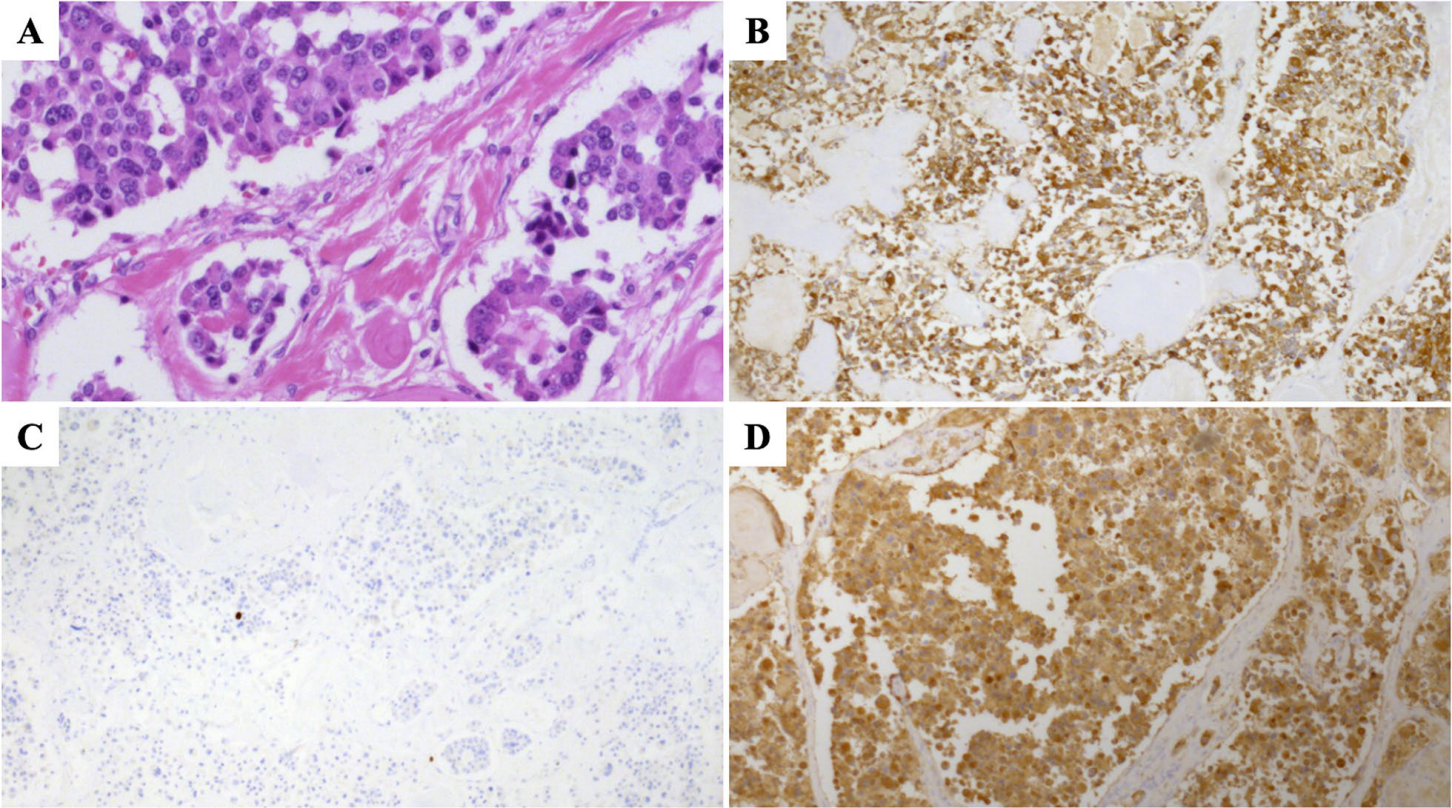
Histologic examination shows neuroendocrine tumors G1 in the liver. **A** Hematoxylin–eosin staining. **B** Positive for chromogranin-A. **C** Ki-67 < 1%. **D** Positive for calcitonin

We performed a left trisectionectomy with a bile duct resection. The operative time was 477 min and the blood loss was 1390 ml. He was discharged to home on postoperative day 8 without complications. The formalin-fixed liver specimen had 3 yellow-white masses (Additional file 1: Figure S1). The histologic examination showed neuroendocrine tumors G1 (Fig. 2). Calcification was not observed. There was no lymphatic, vascular, or bile duct invasion. Lymph node metastasis was not observed. Chromogranin-A and synaptophysin were positive. Carcinoembryonic antigen and thyroid transcription factor 1 were weakly positive. Immunohistochemistry was strongly positive for calcitonin, but negative for insulin, gastrin, glucagon, somatostatin, and vasoactive intestinal polypeptide. The serum calcitonin level was elevated (389 pg/ml). Thus, we diagnosed calcitonin-producing neuroendocrine neoplasms of the liver.

**Fig. 3:**
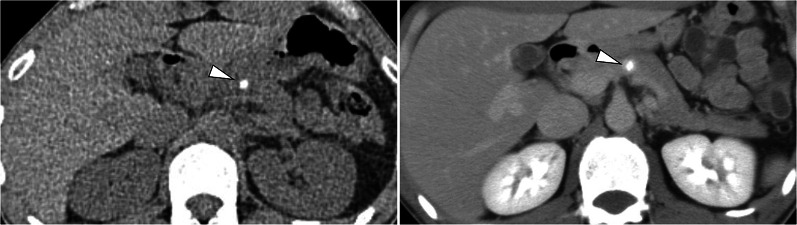
Computed tomography shows a 4-mm calcification (**A**) but no contrast-enhanced mass (**B**) in the pancreatic body

Neuroendocrine neoplasms of hepatic origin are extremely rare, so a systemic exploration was performed. We performed upper and lower endoscopy, a thyroid gland ultrasonography, 18F-fluorodeoxyglucose positron emission tomography (FDG-PET), and somatostatin receptor scintigraphy, but no tumor was identified. We re-reviewed the preoperative CT, which showed a 4-mm calcification in the pancreatic body, but no contrast-enhanced mass was observed (Fig. 3). There was no evidence of tumor in the calcified area, so we followed the patient closely.

**Fig. 4:**
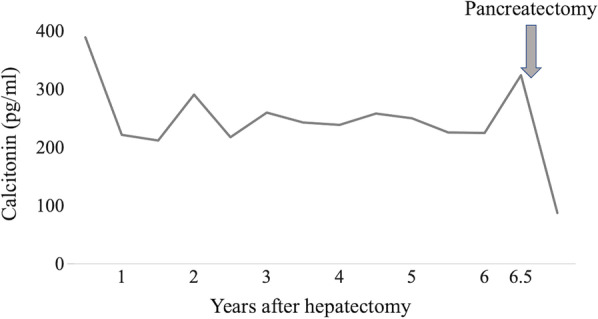
Serum calcitonin levels after hepatectomy

**Fig. 5:**
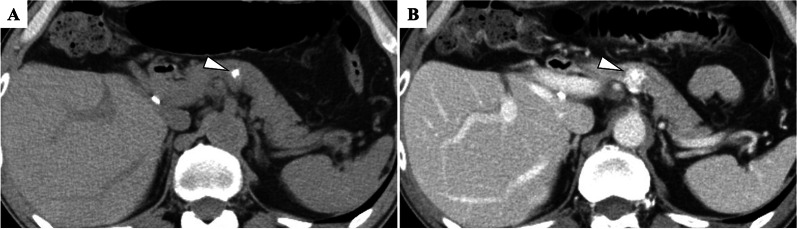
Computed tomography shows a 4-mm calcification (**A**) and a 10-mm contrast-enhanced mass (**B**) in the pancreatic body

**Fig. 6:**
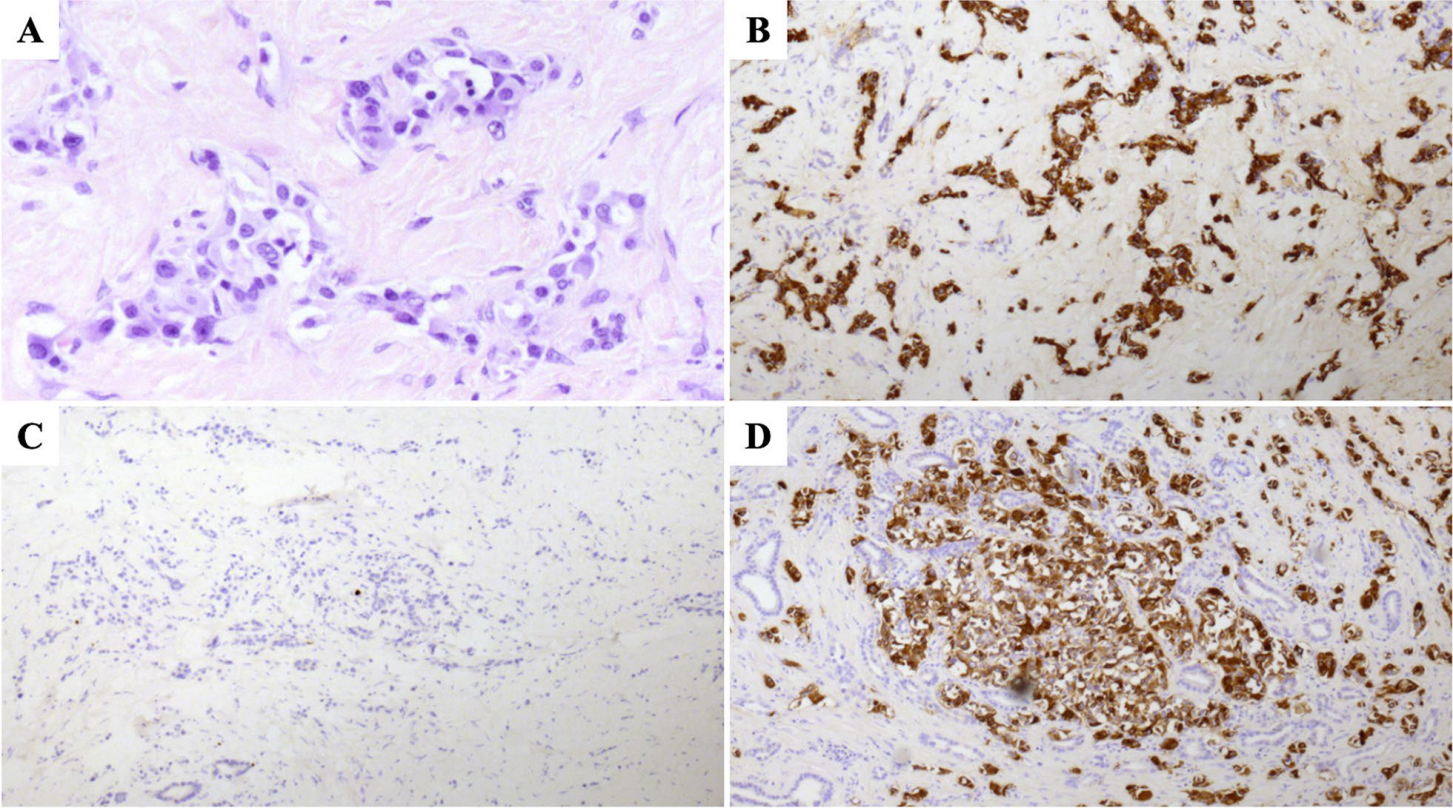
Histologic examination shows a neuroendocrine tumor G1 in the pancreas similar to the liver tumors. **A** Hematoxylin–eosin staining. **B** Positive for chromogranin-A. **C** Ki-67 < 1%. **D** Positive for calcitonin

Although the liver tumors were resected, diarrhea persisted and the serum calcitonin also continued to be elevated (Fig. 4). Diarrhea had occurred 3–5 times a day. Repeat ultrasonography, CT, FDG-PET, and somatostatin receptor scintigraphy were performed for 6 years after the hepatectomy, but no obvious tumor was identified; however, 6.5 years after the hepatectomy, laboratory testing revealed elevation of the serum calcitonin level (324 pg/ml). We performed a CT, which showed a 10-mm contrast-enhanced mass in the calcified area of the pancreatic body (Fig. 5). FDG-PET was performed, but showed no significant accumulation. The tumor was suspected to be a PanNEN, thus we performed a distal pancreatectomy. The operative time was 285 min and the blood loss was 509 ml. He was discharged to home on postoperative day 8 without complications. The formalin-fixed pancreatic specimen showed a yellow-white mass that mimicked the liver tumors (Additional file 2: Figure S2). The histologic examination revealed a neuroendocrine tumor G1 that mimicked the liver tumors (Fig. 6). Calcification 4 mm in size was observed. Minimal lymphatic, arterial, and venous invasion was observed, but perineural invasion was not observed. Lymph node metastasis was not observed. Chromogranin-A and synaptophysin were positive. Carcinoembryonic antigen and thyroid transcription factor 1 were also weakly positive. Immunohistochemistry was strongly positive for calcitonin, but negative for insulin, gastrin, glucagon, somatostatin, and vasoactive intestinal polypeptide. After a distal pancreatectomy, the serum calcitonin level decreased and diarrhea resolved. We finally diagnosed him as a calcitonin-producing PanNEN with liver metastases (pathological T1N0M1, stage IV according to the Union for International Cancer Control classification of malignant tumors, 8th edition) [5]. He is currently recurrence-free 6 months after the distal pancreatectomy.

## Discussion

Calcitonin-producing PanNENs have been reported to have synchronous liver metastases in approximately 50% of cases, but there are no reports in which liver metastases are the presenting symptom [2, 6–36]. In the present case, however, calcitonin-producing neuroendocrine neoplasms in the liver were evident first, followed by a PanNEN 6.5 years later. A neuroendocrine neoplasm of hepatic origin is extremely rare, while the liver is the most common metastatic site for PanNENs [37–39]. In the present case, high serum calcitonin levels and diarrhea persisted after the hepatectomy, but after a distal pancreatomy the serum calcitonin level decreased and diarrhea resolved. These findings suggest that a micro-PanNEN was present in the calcified area in the pancreatic body from the beginning and manifested over a period of 6.5 years. Therefore, it was reasonable to consider that a calcitonin-producing neuroendocrine neoplasm was a pancreatic primary and hepatic tumors were metastases. The present case demonstrated that a calcitonin-producing PanNEN may be initially recognized as a liver tumor that may become evident after a long period of time. Therefore, careful long-term observation is necessary when a calcitonin-producing neuroendocrine neoplasm is observed in the liver.

Although micro-calcifications are often seen in PanNENs, coarse calcifications are extremely rare [3, 40]. The present case revealed that calcitonin-producing PanNENs may show coarse calcifications. Although CT showed a coarse calcification in the pancreatic body, no tumor was detected for a long period of time. Furthermore, FDG-PET and somatostatin receptor scintigraphy showed no accumulation in the coarse calcified area. Therefore, it should be considered that calcitonin-producing PanNENs may be present in coarse calcified areas in the pancreas, even in the absence of findings other than coarse calcifications. It is difficult to decide whether to perform pancreatectomy for the coarse calcified area at the time of hepatectomy, when contrast-enhancing tumors are not apparent because simultaneous hepatectomy and pancreatectomy are high-risk procedures [41]. A watch-and-wait strategy may be acceptable for cases with coarse calcifications but no contrast-enhanced masses in the pancreas.

The main symptom of calcitonin-producing PanNENs is diarrhea, which appears in more than half of patients [2]. Calcitonin is usually secreted by the C cells of the thyroid, but very rarely by PanNENs [2, 25]. Calcitonin reduces serum calcium levels as an antagonist of parathormone, and is also associated with intestinal interactions, inhibiting gastrin and gastric acid secretion and increasing sodium, potassium, chloride, and water secretion, which may explain appearing diarrhea in patients with the high serum calcitonin level [2, 9, 25, 42, 43]. In addition, diarrhea resolves when the serum calcitonin level decrease by resection of calcitonin-producing tumors [2, 44–46]. The present case also had suffered from diarrhea for a long time, but diarrhea resolved by resection of a calcitonin-producing PanNEN and decreasing the serum calcitonin level.

Surgical resection is the only curative treatment for calcitonin-producing PanNENs with liver metastases, as with other PanNENs [2, 4]. The present case underwent highly invasive surgeries with a left trisectionectomy, followed by a distal pancreatectomy, both of which had good postoperative outcomes and may have contributed to the long-term prognosis. These favorable outcomes may be attributed to surgeries performed by a board-certified expert surgeon from the Japanese Society of Hepato-Biliary-Pancreatic Surgery [47]. It is considered important that highly advanced surgery should be performed by board-certified expert surgeons.

## Conclusions

Calcitonin-producing PanNENs may be initially recognized as liver tumors and may become evident after a long period of time, so careful long-term observation is necessary. If complete resection is possible, aggressive surgeries may contribute to a long-term survival for patients with PanNENs.

## Supplementary Information

**Additional file 1: Figure S1.** Gross appearance of the liver shows yellow-white masses in segment 1 (A) and segment 4 (B, C). MHV, middle hepatic vein; LPV, left portal vein; RAPV, right anterior portal vein; CHD, common hepatic duct.

**Additional file 2: Figure S2.** Gross appearance of the pancreas shows a yellow-white mass similar to the liver tumors.

## Data Availability

The data used for this case report are available from the corresponding author upon request.

## Abbreviations

PanNEN: Pancreatic neuroendocrine neoplasm
CT: Computed tomography
FDG-PET: 18F-fluorodeoxyglucose positron emission tomography
