# Supplementary figures and images for: A calcitonin-producing pancreatic neuroendocrine neoplasm treated with distal pancreatectomy a lengthy time after a left trisectionectomy for liver metastases: a case report
Source: Surg Case Rep. 2022 Dec 8;8:217. doi: 10.1186/s40792-022-01575-7 (PMC9732168; doi:10.1186/s40792-022-01575-7)

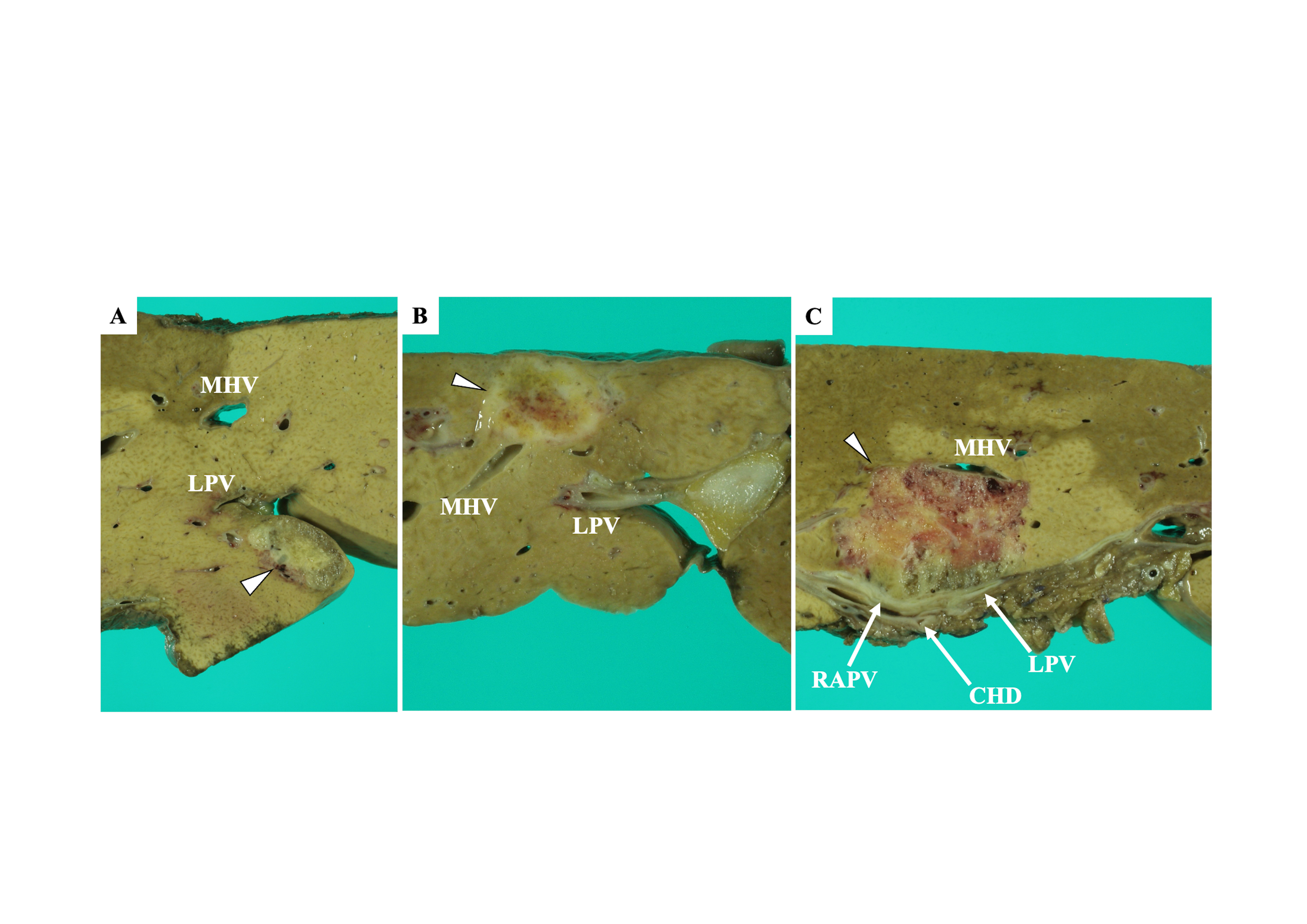

Supplement: Supplementary file 1 — Additional file 1: Figure S1. Gross appearance of the liver shows yellow-white masses in segment 1 (A) and segment 4 (B, C). MHV, middle hepatic vein; LPV, left portal vein; RAPV, right anterior portal vein; CHD, common hepatic duct. [file 40792_2022_1575_MOESM1_ESM.jpg]

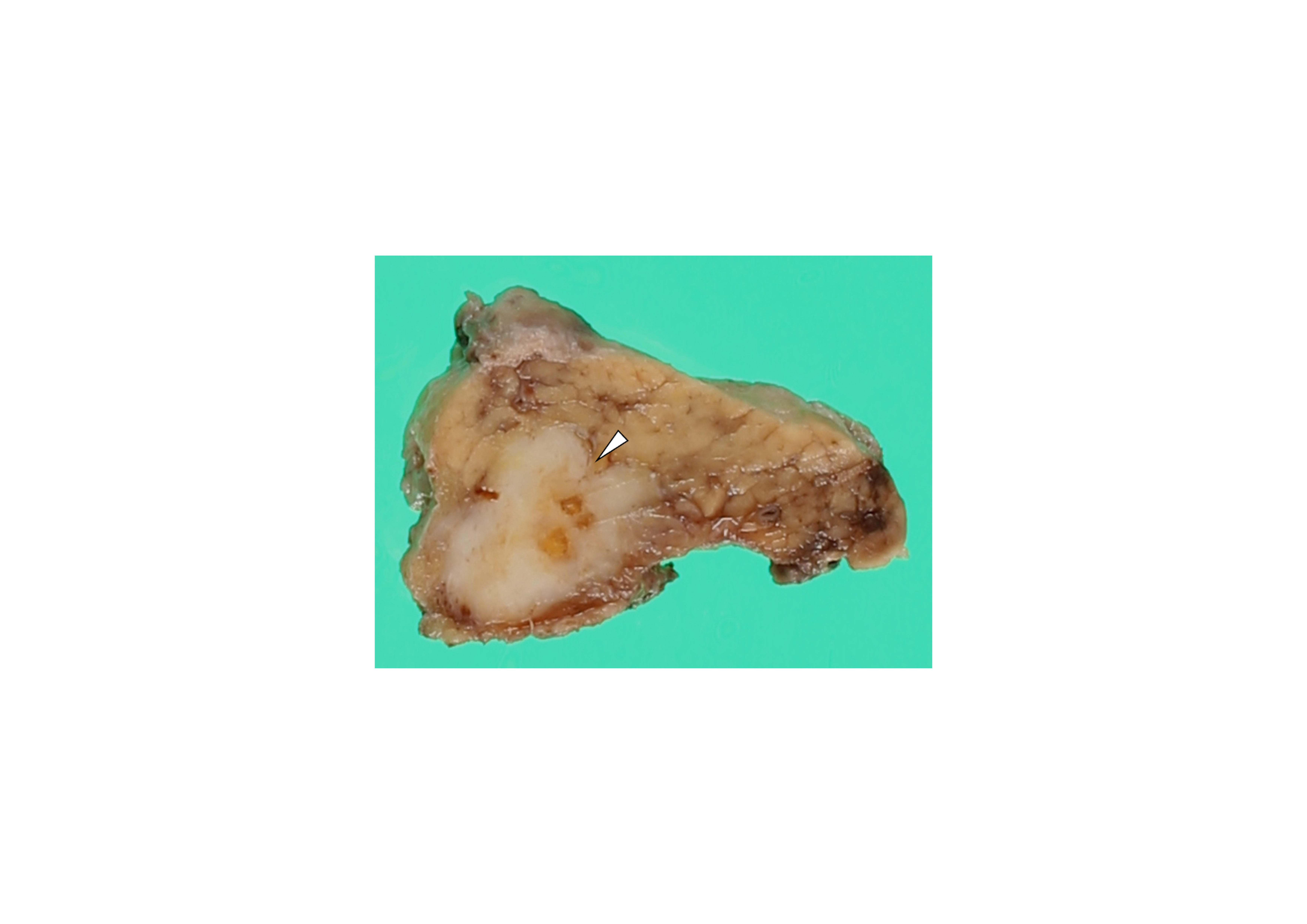

Supplement: Supplementary file 2 — Additional file 2: Figure S2. Gross appearance of the pancreas shows a yellow-white mass similar to the liver tumors. [file 40792_2022_1575_MOESM2_ESM.jpg]
